# Microstructure and phase evolution in increasing the amount of mo and Ti of non-equiatomic CoFeNi-based medium entropy alloys for organic wastewater treatment

**DOI:** 10.1038/s41598-025-03976-8

**Published:** 2025-05-29

**Authors:** Emőke Sikora, Ferenc Kristály, Dora Tergalecz, Anna Sycheva, Tibor Ferenczi, Maria Sveda, Dora Janovszky

**Affiliations:** 1 Institute of Chemistry, University of Miskolc, Miskolc, Hungary; 2 Department of Mineralogy and Petrography, University of Miskolc, Miskolc, Hungary; 3 HUN-REN-ME Materials Science Research Group, University of Miskolc, Miskolc, Hungary; 4 Institute of Energy, Ceramics and Polymer Technology, University of Miskolc, Miskolc, Hungary; 5 Institute of Foundry and Metallurgy, University of Miskolc, Miskolc, Hungary; 6 Institute of Physical Metallurgy, Metalforming and Nanotechnology, University of Miskolc, Miskolc, Hungary

**Keywords:** High entropy alloys, Mechanical alloying, Dye decolourisation, Rhodamin B, Materials science, Materials for energy and catalysis, Structural materials

## Abstract

The effect of the combined addition of Mo and Ti on the phase evolution of non-equiatomic (CoFeNi)_100−2.5x_Mo_x_Ti_1.5x_ (x = 2,4,6,8,10,12) alloys produced by high-energy ball-milling was investigated. Based on the preliminary phase stability criteria, solid solution formation is expected for each composition, a face-centred structure (FCC) phase if x is less than 10, and FCC and body-centred structure (BCC) phase in other cases. After 35 h of milling, solid solution structures were successfully produced in all samples; an FCC, two BCC structure phases, and a small amount of Co phase were identified based on X-ray diffraction. One of the BCC phases is Mo-based (BCC1(Mo)), while the other is a Fe-based (BCC2(Fe) solid solution. Increasing the combined amount of Mo and Ti alloying up to 20 at% (x = 8), the amount of the FCC structure was dominant, while above 20 at% (x = 10), the amount of the two BCC lattice structures was predominant. The average particle sizes were smaller than 3 μm. The specific surface area of all composition powders was less than 0.25 m^2^/g, which is extremely rare for a catalyst. All HEA powders containing Mo and Ti demonstrated enhanced photocatalytic activity in the decolorisation of Rhodamine B dye (RhB). The optimum conditions for RhB decolorisation were a pH of 2 and a catalyst dosage of 1 g/L. Under these conditions, (CoFeNi)_85_Mo_6_Ti_9_, (CoFeNi)_80_Mo_8_Ti_12_, and (CoFeNi)_70_Mo_12_Ti_18_ demonstrated high efficiencies of 97.6, 98.6 and 98.7%, respectively, already in the first minute of reaction.

## Introduction

Multicomponent high-entropy alloys (HEAS) have garnered significant attention since their initial discovery. Both equimolar and non-equimolar variants of HEAs can be synthesized, with their crystal structures, especially in compositions containing five or more elements, being influenced by various factors beyond configurational entropy^1^. These alloys may adopt a single-phase solid solution, such as face-centred cubic (FCC)^2^, body-centred cubic (BCC)^3^, or hexagonal close-packed (HCP)^4^ structures. However, it is also common for HEAs to exhibit a complex mixture of phases, including intermetallic and amorphous structures. Among these, FCC-type solid solution HEAs constitute a substantial portion³.

Certain elements can destabilase the FCC structure, while others promote BCC formation or encourage the development of intermetallic phases. It shoud be noted that CoFeNi-based HEAs, are frequently studied due to their favourable magnetic properties^2^^,5^ and their tendency to form stable FCC structures. Previous studies have investigated the phases present in CoFeNi-containing HEA powders, which are summarised in Table 1. Several equiatomic HEAs—such as CoCrFeNi^5-7^ CoCrFeNiMn^5^^,6^, CoFeNiV^8^, CoFeNiMn^9^, and AlCoFeNiTi^10^—have been confirmed to form single-phase FCC solid solutions.

Despite aluminium (Al) having an FCC lattice in its pure form, its incorporation into CoFeNi HEAs promotes the formation of a BCC structure^2^^,11^^,12^. At a 0.5 molar ratio, the BCC phase becomes evident, and at equimolar concentration, the alloy transforms entirely to a BCC phase¹¹. Additionally, elements such as Si^11^ and Ti^13-16^, even in non-equiatomic amounts, tend to form intermetallic phases with Ni. The inclusion of Mo^8^^,17^ in CoFeNi HEAs also facilitates the formation of intermetallic structures. Verma et al.^18^ reported the coexistence of FCC and BCC phases following 36 h of milling in HEAs containing equimolar ratios of Al and Mo.

In parallel with the advancement of HEA research, environmental concerns are also prompting the exploration of novel materials for water treatment. The value of clean water is rising globally, with industrial activities placing increasing demand on water resources and intensifying the importance of wastewater management.

 In the current study, Rhodamine B (Rhb) was used as a model pollutant to simulate wastewater solution. Rhodamine B (RhB) is a synthetic, water-soluble fluorescent dye, classified as one of the most important xanthene dyes^19^ It is commonly used in the dyeing of cotton, bamboo, leather, weed, fireworks, and even ballpoint pens, leading to its release into the environment. RhB dye poses several risks to aquatic life, including respiratory damage, tissue necrosis, reproductive harm, and even cancer. Additionally, RhB is highly stable and difficult to biodegrade, which contributes to its long-term environmental pollution^20^.

To address this, researchers are investigating cost-effective and efficient water purification technologies. Wang et al.^21^ demonstrated the rapid degradation of azo dyes using Fe-based metallic glasses, attributing the reactivity of these materials to their metastable thermodynamic state and surface morphology. Given the thermodynamic metastability and diverse elemental composition of HEA powders, these materials are also being explored for environmental applications. Notably, Lv et al.^22^ reported in 2012 the successful degradation of azo dyes using AlCoCrTiZn HEA powder, highlighting the potential of HEAs in wastewater treatment.

In this study, the role of the combined effect of Mo and Ti on the amount of phases formed in CoFeNi HEA and its catalytic activity was investigated in detail.

**Table 1:** Phases of different CoFeNi contain HEAs.

Samples	Phases	Year, Reference,
CoFeNi	FCC	2019, ^5^
CoCrFeNi	FCC	
CoCrFeMnNi	FCC	
Al_0.5_CoFeNi	FCC + BCC	2014,^11^
AlCoFeNi	BCC	
CoFeNiSi_0.25_	FCC	
CoFeNiSi_0.5_	FCC + Ni_3_Si	
(CoFeNi) _80_ Ti_5_V_15_	FCC + L1_2_	2023,^13^
(CoFeNi)_84_Ti_8_V_8_	FCC + L1_2_ L1_2_:(Ni, Co, Fe)_3_(Ti, V) fcc a = 0,3596 nm	2022, ^14^
(FeCoNi)_94_Ti_6_	FCC + Ni_3_Ti	2020, ^15^
FeNiCoMn	FCC	2014, ^9^
FeNiCoV	FCC	2015, ^8^
FeNiCoVMo_x_ x:0.2, 0.4–1	FCC + CoMo_2_Ni Co_2_Mo_3_-type ICDD: 00–009-0298, R-3 m, Cubic	
Co_25_Fe_25_Mn_5_Ni_25_Ti_20_	FCC + BCC+ Ti_2_Ni + Ti_2_Co	2020, ^16^
CoCrFeNi and CoCrFeMnNi	FCC+	2019, ^6^
CrCoFeNi	FCC	2019, ^7^
AlCoFeNiTi	BCC + FCC	2015, ^10^
AlCoFeNiMo	BCC + FCC	2024, ^18^

## Materials and methods

### Preparation of HEA powders

High-purity powders of Co (99.8% pure, 150–45 μm size), Fe (99% pure, < 75 μm size), Ni (99.8% purity, 100–75 μm size), Ti (99.4% pure, < 150 μm size), Mo (99.95% pure, 3–7 μm size) have been used in this work. The non-equiatomic (CoFeNi)_100−2.5x_(Mo_x_Ti_1.5x_) alloy compositions were prepared (x = 2, 4, 6, 8, 10, 12). HEA powders were referred to as Mo_2_Ti_3_, Mo_4_Ti_6_, Mo_6_Ti_9_, Mo_8_Ti_12_, Mo_10_Ti_15_, and Mo_12_Ti_18_ respectively. Subscripts in the alloy compositions indicate the atomic portion of each element.

These powders were ball-milled in an argon atmosphere in a planetary ball mill (Fritsch, Pulverisette 5) using a 250 ml hardened steel vial (63 HRC). Balls were selected from hardened steel. A combination of balls with different diameters was used for milling: 5 balls with 20 mm diameter, 14 balls with 12 mm diameter, 4 balls with 10 mm diameter and 10 balls with 7 mm. Given that Fe is already present in the composition, the effects of possible Fe transportation from balls to powders on the mechanical properties are marginal. The ball-to-powder ratio was 13:1, and the milling speed was 200 rpm. Each one-hour milling process was followed by 1 h to cool down the vials. Toluene was used as a process control agent (10 ml).

### Phase stability

Multicomponent (minimum 4, 5) phase diagrams are not generally available. Therefore, Zhang et al.^4^, Guo et al.^23^ and Yang et al.^24^ have developed empirical criteria for predicting solid solution formation to design new HEA alloys. These criteria have been established based on the phases of the HEA alloys found in the literature. The calculated parameters according to the requirements are presented in Table 2. For each composition, a solid solution is expected to form based on all three criteria; in the case of (Mo_10_Ti_15_) and Mo_12_Ti_15_, FCC and BCC lattices are expected, while in the other cases, only the FCC lattice phase is expected.

**Table 2:** The calculated values of parameters (ΔH_mix_, ΔS_mix_, Δ, Ω, VEC) for the investigated alloys.

Parameter	Designed value	Mo_2_Ti_3_	Mo_4_Ti_6_	Mo_6_Ti_9_	Mo_8_Ti_12_	Mo_10_Ti_15_	Mo_12_Ti_18_
Mixing enthalpy* (kJ/mol)	−16≤ ∆H_mix_≤5	−4.6	−7.6	−10.2	−12.4	−14.4	−16.0
Mixing entropy	1.32≤∆S_mix_≤2.35 R	1.28	1.38	1.46	1.51	1.55	1.58
Atomic size mismatch (δ)	0 ≤ δ ≤ 8.5	3.2	4.37	5.19	5.77	6.22	6.56
Ω	Ω ≥ 1.1	4.14	2.77	2.19	1.90	1.71	1.59
Valence electron concentration (VEC)	6.87 BCC ≤ VEC ≤ 8 FCC	8.67	8.58	8.37	8.16	7.95	7.74

^*^ The binary mixing enthalpies in ternary amorphous systems used in the calculation can be found in Ref^23^ IM -intermetallic, SS- solid solution.

### Materials characterisation

Bruker D8 Advance diffractometer (XRD) was utilised to analyse phase structure using a diffractometer using Cu Kα radiation (40 kV, 40 mA) in parallel beam geometry obtained with a Göbel mirror equipped with a Vantec-1 position sensitive detector (1° window opening), measured in the 2–100 °(2 θ) angular range, at a 0.007° (2 θ)/29-sec speed. The specimen was rotated in the sample plane during the measurement to obtain data from the whole surface and reduce in-plane preferred orientation effects. The quantitative results were obtained by the combined use of Rietveld refinement and peak area calculation (Pawley fit). The amorphous fraction was determined using peak area determination from the Pawley fit in TOPAS4 (amorphous hump method). Crystallite sizes were calculated from peak broadening by Scherrer equation after correction for instrumental contribution, during the Rietveld refinement. The instrumental profile was calculated by the empirical parametrisation method using NIST 640 d Si powder as a standard. The background was fitted by the use of Chebyshev polynomial 4 th degree and hyperbola (1/x) function for air scattering at low angles. For each phase, a fitting pattern was calculated from initial unit cell values and atomic coordinates from ICSD database or relevant literature. During the refinement unit cell parameters and crystallite sizes were fitted to estimate the actual values. Due to the parallel beam geometry, sample displacement, surface roughness and other errors were eliminated and needed no correction, also making calculation errors minimal. The peak broadening was resolved by simultaneous size and strain calculation, size (nm) = FWHM(2θ)*cos(θ)/λ and strain ε0 = FWHM(2θ)/(4 tanθ, a dimensionless parameter which could be related to the distortion of the lattice parameter in percent).

The weight fractions of phases were determined using the Rietveld method and then converted to volume fractions using the phase density. The microstructure of the powders was analysed by a Scanning Electron Microscope (Thermo Scientific Helios G4 PFIB SEM) equipped with an Energy Dispersive Spectrometer (EDS). After 35 h of milling, the composition of the alloys was controlled by SEM-EDS analysis. The specific surface area of the HEA powder was examined using the Brunauer–Emmett–Teller method (BET, Micrometrics TriStar 3000). Nitrogen adsorption-desorption isotherms were acquired at 77 K using. The particle size distributions were done on the SEM images based on measuring 500 particles using Fiji ImageJ 2.16.0 version software (https://fiji.sc).

### Catalytic activity measurements

The decolourisation of RhB (C_28_H_31_CIN_2_O_3_, Fluka AG) was evaluated under UV light irradiation experiments on the HEA samples. In every experiment, three parallel reaction mixtures were prepared as follows: 250 mg sample was added for 250 mL of 5 mg/L RhB solution. The HEA powder was treated in 1 M HCl solution for 10 min before adding to the RhB solution. To test the pH dependence, the pH of the solution was adjusted with 1 M NaOH and 1 M HCl solutions using a digital pH meter. After these preparations, the samples were stirred on a magnetic stirrer for 15 min to disperse the catalyst. In order to check if adsorption would occur and if the dye would be bound by the HEA, the concentration of the RhB was measured. Negligibly small concentration differences were detected, so the adsorption did not take place during the process. After that, 1,25 ml of hydrogen peroxide solution (H_2_O_2_, 50 wt %) was injected into the solutions. The mixing was continued for a further 20 min, with sampling. Samples were taken at 1, 2, 5, 8, 12, 15 and 20 min from each beaker. The samples were filtered through a syringe filter and analysed using a UV-visible spectrophotometer on 553 nm. At certain time intervals, 1–1 sample was taken from the magnetic stirrer and has been filtered. The RhB concentration of the filtrates was determined using an EMITA VP-60 UV lamp (power 180 W). It transmits UV radiation in the range of 320–400 nm; the maximum emission was at 365 nm wavelength.

## Results and discussion

### Morphology of milled powder

**Fig. 1:**
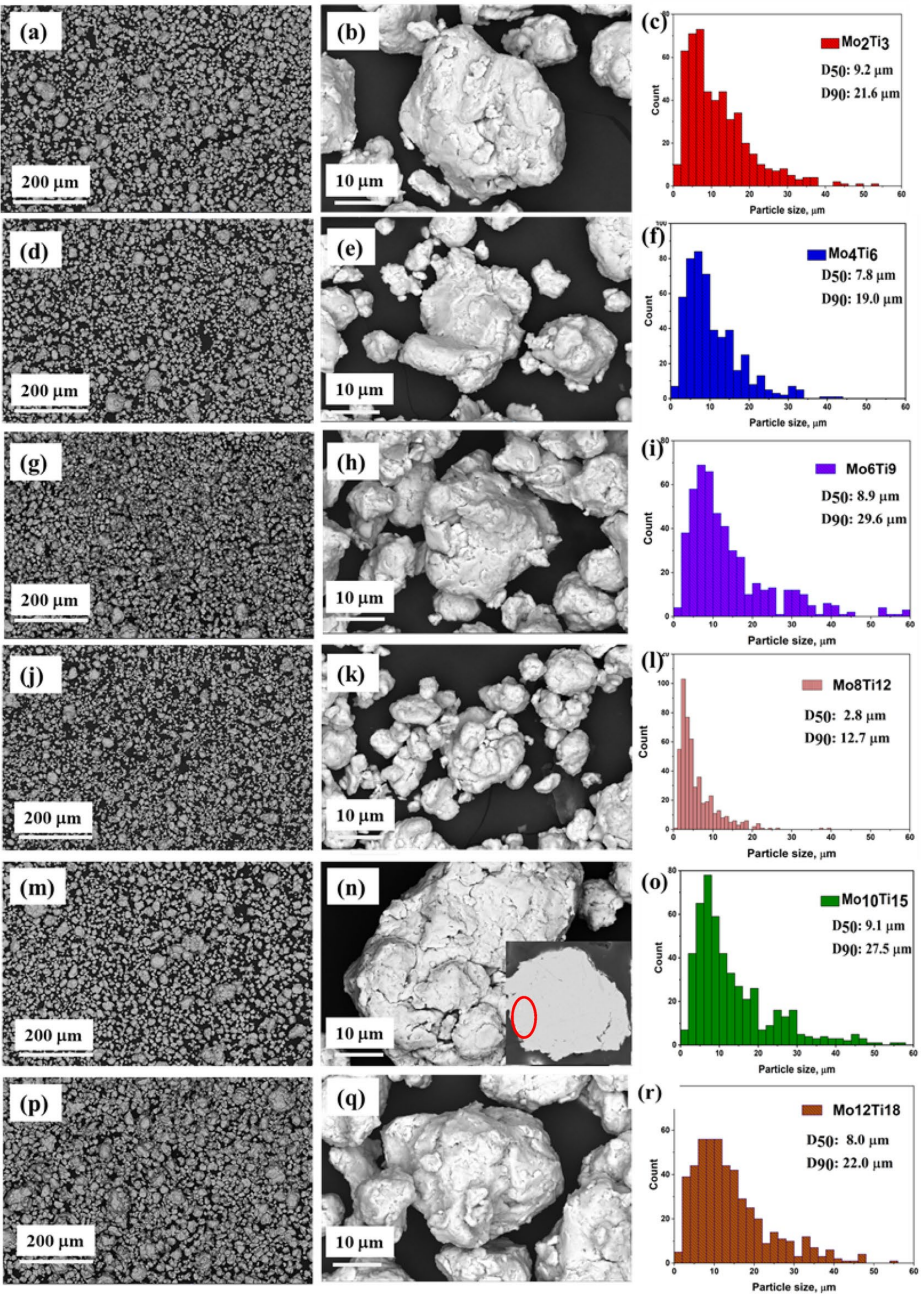
SEM images and particle size distributions of Mo_2_Ti_3_ powders (**a**,** b**,** c**), Mo_4_Ti_6_ (**d**,** e**,** f**), Mo_6_Ti_9_ (**g**,** h**,** i**) Mo_8_Ti_12_ (**j**,** k**,** l**) and Mo_10_Ti_15_ (**m**,** n**,** o**) powders milled for 35 h.

The morphology and microstructure changes with particle size distribution were examined using SEM analysis in (CoFeNi)_100−2.5x_(Mo_x_Ti_1.5x_) alloy powder produced by 35 h high energy milling time alloy powder (Fig. 1). The low-magnification images showed that, in addition to the many tiny particles, larger particles are also visible due to the cold welding between the particles as a result of plastic deformation (see Fig. 1a, d,g, j,m, p). It can be seen in histograms as the D90 value proposes the size of larger particles from 12.7 μm to 29.6 μm and include rare particles with a higher size. As a consequence of continuous fractures and cold welding, the shape of the powder approaches visually spherical, regardless of Mo and Ti content, as seen in Fig. 1b, e,h, k,n, q. As shown in magnified SEM images, smooth parts on the surface of the powders can be seen, suggesting a small specific surface area value. Only a small amount of elemental Co could be distinguished in the cross-section of the embedded powders in SEM backscattered electron images (marked with a red circle), with no apparent colour differences in the other areas, indicating a homogeneous distribution of elements (see Fig. 1n insert picture).

### Structure analysis

**Fig. 2:**
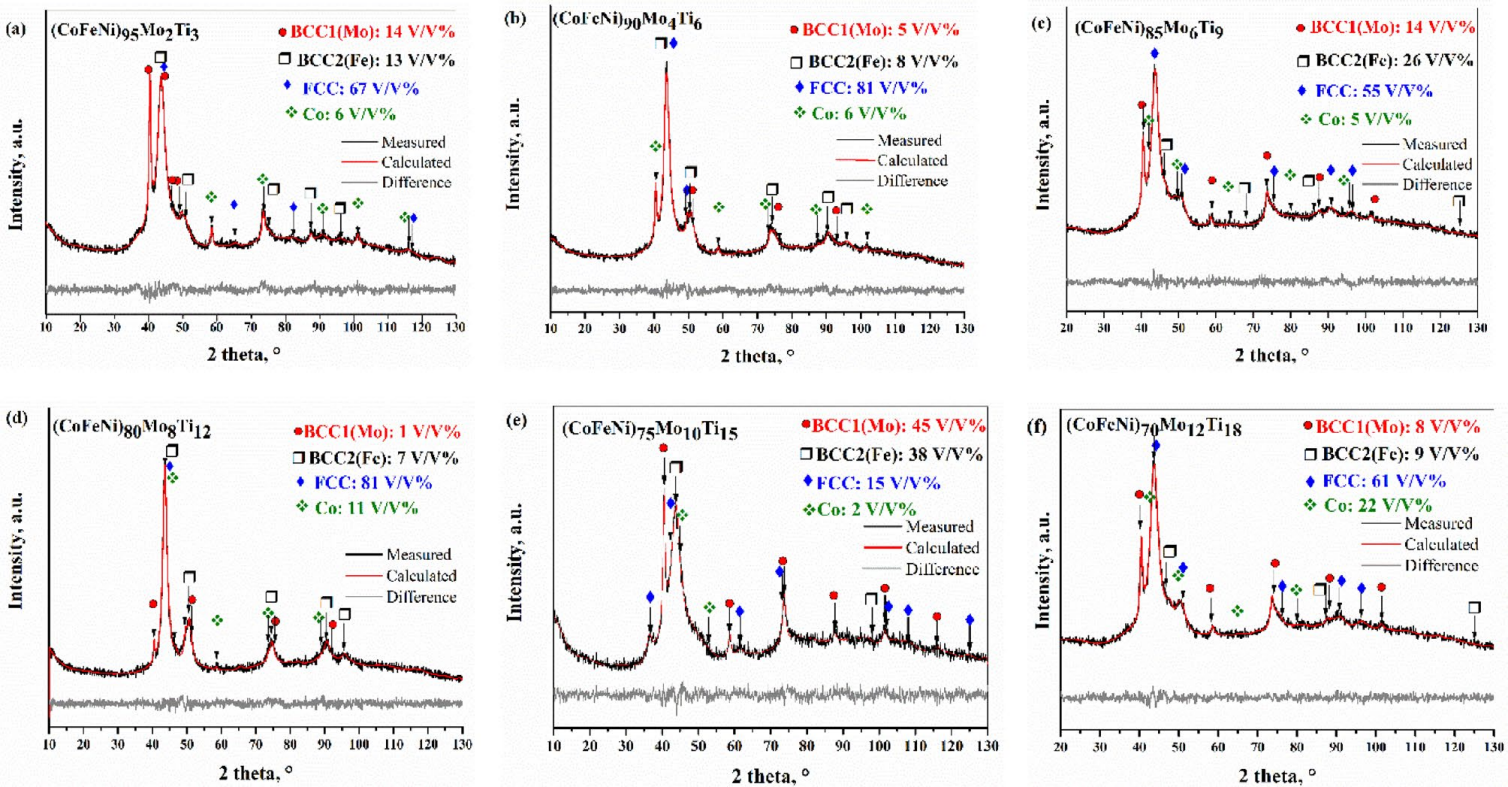
Rietveld refinement of diffraction patterns of (CoFeNi)_100−2.5x_(Mo_x_Ti_1.5x_) powder after 35 h milling time: (**a**) (CoFeNi)_95_Mo_2_Ti_3_, (**b**) (CoFeNi)_90_Mo_4_Ti_6_, (**c**) (CoFeNi)_85_Mo_6_Ti_9_, (**d**) (CoFeNi)_70_Mo_8_Ti_12_, (**e**) (CoFeNi)_75_Mo_10_Ti_15_, (**f**)) (CoFeNi)_70_Mo_12_Ti_18_.

The X-ray diffraction pattern of (CoFeNi)_100−2.5x_(Mo_x_Ti_1.5x_) powders, together with calculated weight fraction and crystallite size after 35 h milling time, are presented in Fig. 2(a-f) and Table 3. Based on the literature, CoFeNi medium entropy alloy has a stable FCC phase^6,11,26^. As can be seen in Fig. 2(a), the Mo_2_Ti_3_ powder consisted mainly of an FCC (Fm-3 m) structure with 64 wt% (67 V/V%). The lattice parameter of this FCC structure was 0.35820 nm. The lattice parameter of the FCC phase of CoFeNi produced by casting is 0.35990 nm^11^, and 0.3648 nm^27^ produced by mechanical alloying. According to thermodynamic calculations, a phase with FCC structure should have been formed only, but two BCC (Im-3 m) structures were also identified. The lattice parameter of the BCC 1 phase with 16 wt% (14 V/V%) was 0.31478 nm, which suggests that this is a Mo-based phase (named BCC 1(Mo)). In the literature, the Mo and Ti element is considered to be the most effective alloying agents that help form an intermetallic phase^8,26,28^. Still, no intermetallic phase was identified in the family of alloys we studied. The lattice parameter of pure Mo is 0.3147 nm, which does not mean that the BCC 1(Mo) is pure Mo. It shows that in addition to the largest atomic diameter Ti, the smaller diameters Co, Fe, and Ni are also present in the lattice so that the lattice enhancing effect of Ti is balanced out. The lattice parameter of the BCC2 (Im-3 m) structure with 12 wt% was 0.28573 nm, which was very similar to the lattice parameter of pure Fe (a_Fe_=0.2857 nm), named BCC 2(Fe). Adding 32 wt% Co content, 7 wt% hexagonal structure Co content was identified after 35 h milling time. The initial Co powder was a mixture of FCC (Im-3 m) and hexagonal (P63/mmc) lattice structure powders. Sort et al.^29^ demonstrated that in the case of Co, the FCC ⟷ HCP phase transformation occurs several times during high-energy milling. In the previous milling experiments^30^, it was observed that this transformation prevents incorporation of Co into the HEA alloy. The lattice parameters of the detected Co (P63/mmc, a_Co_=0.23967, c_Co_=0.38943) are smaller than those of pure Co (a_Co_=0.25071, c_Co_=0.40695), which means that the smaller atomic radii Fe and Ni may be incorporated in the Co lattice. As shown in Table 3, doubling the amounts of Mo and Ti increased the amount of FCC structure up to 79 wt% (81 V/V%). The quantity of the volume of two BCC structures phases decreased by 40–60%. The variation of the lattice constants is less than 0.5%, except for hexagonal Co, where the parameter “c” increased. Observing Fig. 2c; Table 3, the FCC structure decreased to 55 V/V%, but this phase remained the main phase. The two BCC phases increased.

Figure 2(d) shows the X-ray results of Mo_8_Ti_12_ powder. It has been noted that the FCC phase was still the major phase (81 V/V%). The BCC structures were also observed but had a lower content than in the case of the other alloys. According to Fig. 2(e), in Mo_10_Ti_15_ powder, a significant change occurred in the quantity of formed phases. The volume of the FCC structure phase decreased significantly. In contrast, the volume of BCC structure phases increased; the BCC1(Mo) phase was 53 wt% (45 V/V%), and the BCC2 (Fe) was 27 wt% (38 V/V%). 2 wt% Co was found in the alloying powder after 35 h of milling, featuring FCC structure. In the case of the Mo_12_Ti_18_ sample, the most remarkable result is that the FCC phase has become the main phase again, and the amount of the phase mainly containing Co has increased.

**Fig. 3:**
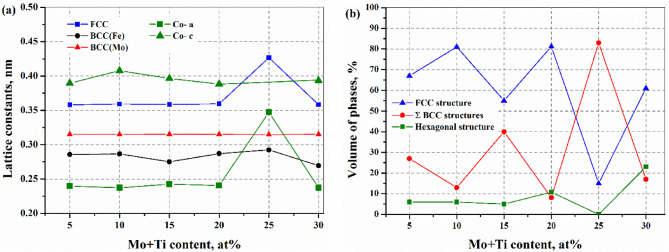
Evolution of lattice parameter (**a**) and volume of different phases (**b**) function of the total Mo and Ti content.

All crystal sizes were below 15 nm owing to the high-energy milling process except the BCC(Mo) (see Table 3). The evolution of the lattice parameters as a function of the total Mo and Ti content can be observed in Fig. 3(a). The lattice parameter of BCC(Fe) increases slightly; in the case of Mo_10_Ti_15_ alloy, the change is 2% for the value measured in the alloy with the lowest Mo and Ti content (Fig. 3(a)). The most significant change is in the lattice parameter of the FCC structure, where it has increased by 19% for Mo_10_Ti_15_ due to the incorporation of larger diameter elements. It is the least amount of FCC phase in this alloy (see Fig. 3(a)). Increasing the combined amount of Mo and Ti alloying to 20 at%, the amount of the FCC structure was dominant, while at 25 at%, the amount of the two BCC lattice structures dominates (Fig. 3(b)).

**Table 3:** Weight fraction and crystallite size in HEAs powder.

Sample		BCC 1 (Mo) Im-3 m		BCC 2 (Fe) Im-3 m		FCC Fm-3 m		Co Fm-3 m/P63/mmc	
	GoF	wt%	crystallite size, nm	wt%	crystallite size, nm	wt%	crystallite size, nm	wt%	crystallite size, nm
(CoFeNi)_95_Mo_2_Ti_3_	0.30	16	16 ± 5	12	5 ± 2	64	4 ± 1	**7**	10 ± 3
(CoFeNi)_90_Mo_4_Ti_6_	0.31	6	17 ± 5	8	5 ± 2	79	6 ± 2	7	15 ± 5
(CoFeNi)_85_Mo_6_Ti_9_	0.28	8	13 ± 4	6	5 ± 1	57	4 ± 1	29	3 ± 1
(CoFeNi)_80_Mo_8_Ti_12_	0.31	2	23 ± 7	6	6 ± 2	79	7 ± 2	13	10 ± 3
(CoFeNi)_75_Mo_10_Ti_15_	0.30	53	13 ± 3	27	4 ± 1	18	5 ± 1	2	11 ± 3
(CoFeNi)_70_Mo_12_Ti_18_	0.26	9	14 ± 4	9	4 ± 1	56	4 ± 1	25	4 ± 1

### Specific surface area

The specific surface area is a fundamental property for the catalytic property. The specific surface area was determined using the Brauner–Emmett–Teller (BET) method. After 35 h of milling, the specific surface area of the powders can be considered below 0.25 m^2^/g (see Table 4). The BET value for usually photocatalytic powders (Bi_24_O_31_Cl_10_:22.55 m^2^/g^31^, MoS_2_:16–32 m^2^/g^32^, ZnCo_2_O_4_:182 m^2^/g^33^, CuO:7.8 m^2^/g^34^) is more than ten times higher, since the higher the BET value, the more active sites are on the surface. The BET values confirm the observation based on the SEM images that the surface of the pores is smooth, with no pores on the micron scale.

**Table 4:** Results of the specific surface area measurements.

Sample	BET, m^2^/g	BJH, m^2^/g	BET pore, nm
(CoFeNi)_95_Mo_2_Ti_3_	0.17	0.782	15.45
(CoFeNi)_90_Mo_4_Ti_6_	0.17	0.083	20.08
(CoFeNi)_85_Mo_6_Ti_9_	0.05	0.032	14.43
(CoFeNi)_80_Mo_8_Ti_12_	0.23	0.129	19.83
(CoFeNi)_75_Mo_10_Ti_15_	0.21	0.129	12.90
(CoFeNi)_70_Mo_12_Ti_18_	0.21	0.131	11.68

### Photocatalytic degradation of RhB dye

**Fig. 4:**
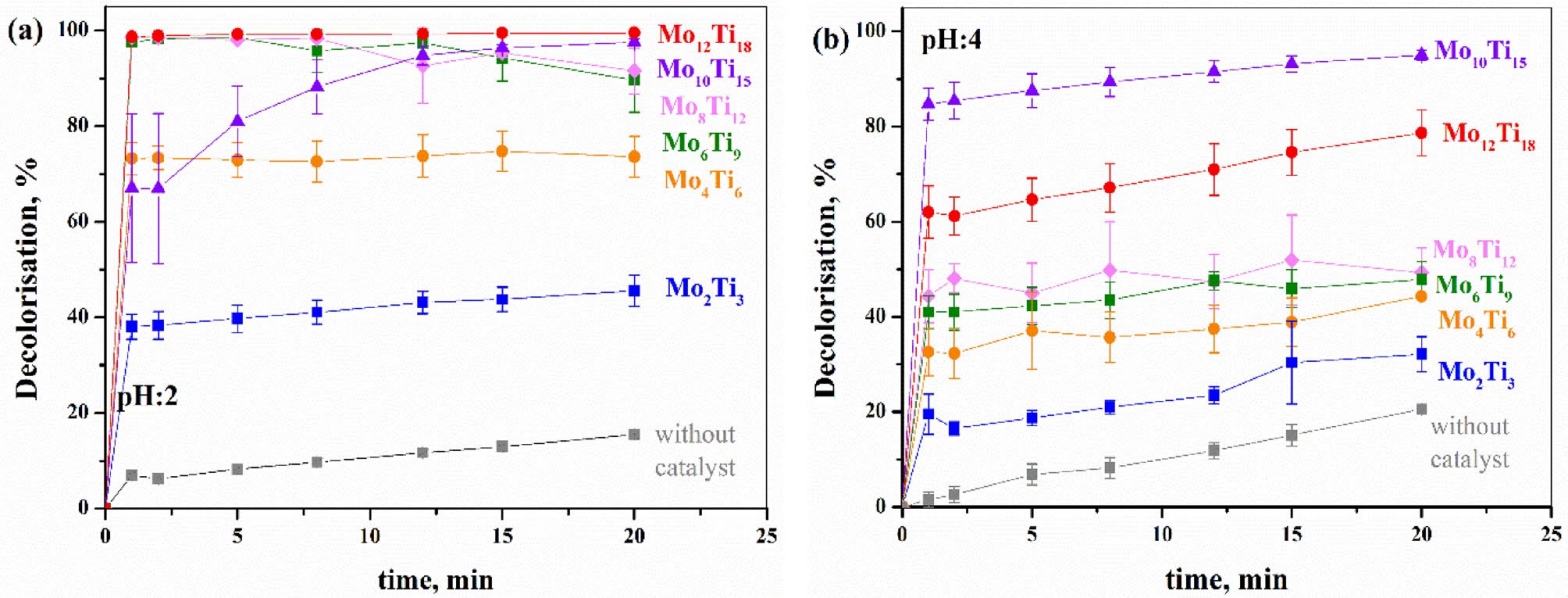
Decolorisation efficiency of RhB by (CoFeNi)_1−2.5x_(Mo_x_Ti_1.5x_) HEA powders under different pH values: pH = 2 (**a**), pH = 4 (**b**).

Figure 4. illustrates the decolourisation of Rhodamine B (RhB) over time. The experiments were conducted both in the presence of HEA powders and without them (grey squares), serving as the reference. The results indicate that the HEA powders significantly accelerated the photo-Fenton oxidation reaction in all cases. Notably, a substantial portion of the dye decolourisation occurred within the first few minutes. As reported in numerous studies^35^, morphology and particle size have a significant impact on catalytic efficiency. In our case, no clear correlation can be established between particle size and catalytic activity, as both the composition ((CoFeNi)_100−2.5x_Mo_x_Ti_1.5x_ (x = 2,4,6,8,10,12)) and particle size of our samples varied (D_50_: 2.8–9.2 μm). However, lowering the pH and the resulting morphological changes have a considerable effect on the processes. Across all experiments, the most effective decolourisation was observed at pH = 2. As Sheng Guo et al.^36^ stated, the pH value has significant influence in case of heterogeneous photo-Fenton reaction processes. On one hand, at lower pH levels, the formation of H_3_O_2_^+^ enhances the efficiency of the degradation process; on the other hand, a rougher or more etched surface typically offers a larger active surface area, providing more reactive sites for dye adsorption and subsequent catalytic degradation^37,38^. The results indicate that increasing the Mo + Ti content generally leads to higher catalytic activity. This trend was observed at both pH values, with one exception. At pH = 4, the Mo_10_Ti_15_ sample degraded 95.0% of the Rhodamine B solution in 20 min, whereas Mo_12_Ti_18_ achieved only 78.7%, contrary to expectations. The results do not indicate strong correlation between crystal size, crystal phases, specific surface area, and catalytic activity. However, it is clear, that increasing the Mo + Ti ratio significantly enhanced the efficiency of the photo-Fenton degradation reaction under these reaction conditions.

**Fig. 5:**
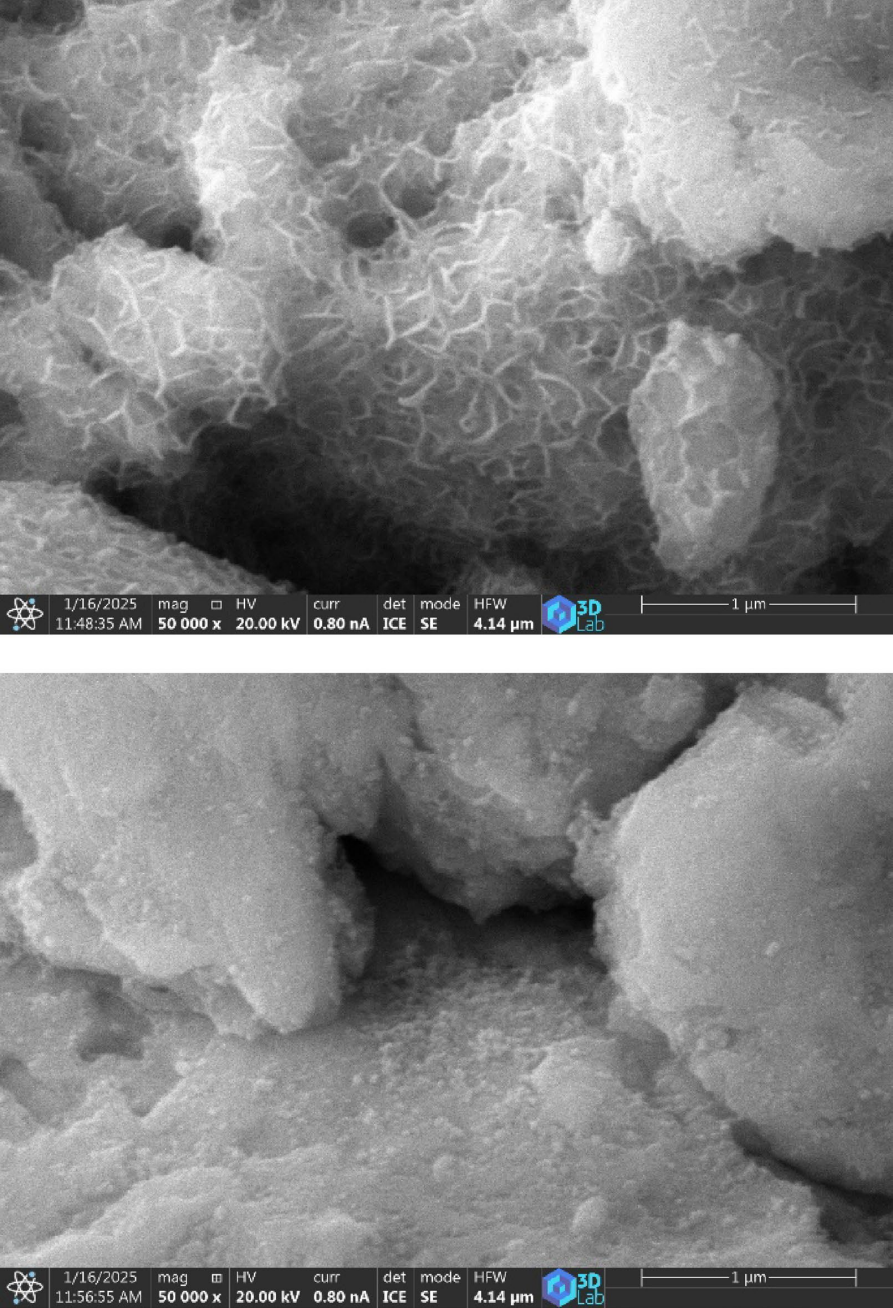
**SEM images of** (CoFeNi)_70_Mo_12_Ti_18_ sample after decolorisation process, using (**a**) pH2 and (**b**) pH4.

Figure 5. shows the surface morphology of the (CoFeNi)_70_Mo_12_Ti_18_ sample after the decolorisation process, using (a) pH2 and (b) pH4, respectively. It can be seen that the etching effect increases surface area of the grains.

Table 5. shows a comparison of the results with some literature data, presenting the highest decolorization rates achieved with the various catalysts, along with the shortest times required to reach them. The comparison showed that the (CoFeNi)_75_Mo_10_Ti_15_ and (CoFeNi)_70_Mo_12_Ti_18_ powders achieved high rhodamine degradation results in a remarkably short time.

**Table 5:** Comparison of our RhB decolorisation results with previously reported literature data.

Catalyst	Reaction condition	Decolourisation rate, %	Decolourisation Time, min	Reference
Fe_2_O_3_-Kaolin under UV light	dye: 15 mg/l catalyst: 1 g/l H_2_O_2_: 0.05 mol/l pH: 2.21	98	120	^36^
ZnO/ZnMoO_4_ visible light	dye: 96 mg/l catalyst: 200 mg/l	83.7	120	^39^
Fe_2_O_3_-Humen acid (adsorption)	dye: 0.5 g/l catalyst: 0.5 g/l pH: 2.53	98.5	15	^40^
Fe_78_Si_9_B_13_ under UV light	dye: 20 mg/L catalyst: 0.5 g pH:3	~ 100	10	^41^
FeCoNiMnCuTi under UV light	dye: 20 mg/l catalyst: 0.97 g/l H_2_O_2_: 1.6 mmol/l pH: 6	88.2	8	^42^
(CoFeNi)_75_ Mo_10_Ti_15_ under UV light	dye: 5 mg/l catalyst: 1 g/l H_2_O_2_: 0.087 mol/l pH: 4	90	8	This work
(CoFeNi)_70_ Mo_12_Ti_18_ under UV light	dye: 5 mg/l catalyst: 1 g/l H_2_O_2_: 0.087 mol/l pH: 2	99	1	This work

## Conclusion

Effects of Mo and Ti content on the microstructure of non-equiatomic (CoFeNi)_100−2.5x_Mo_x_Ti_1.5x_ (x = 2,4,6,8,10,12) HEAs were systematically studied. The conclusions from the experimental results can be summarised as follows:

1. Solid solution HEA alloys have been successfully obtained without inclusion of an intermetallic phase by high-energy grinding for 35 h, even when Mo and Ti both were added based on the XRD studies.
2. Adding Mo and Ti together resulted in the formation of near-spherically shaped particles with an average size varying from 2.8 μm to 9.2 μm particles.
3. The surface of the powders was smooth; the BET value was below 1 m^2^/g in all cases, and the agglomerates had no pores on the micron scale detected.
4. Increasing the combined amount of Mo and Ti alloying to 20 at%, the amount of the FCC structure was dominant, while above 20 at%, the amount of the two BCC lattice structures was predominant. The crystallite sizes were below 25 nm.
5. The decolorisation process efficiency was high even at pH = 4, 90% dye degradation was measured in 8 min using (CoFeNi)_75_Mo_10_Ti_15_ powder. Using pH 2 and a catalyst dosage of 1 g/L, (CoFeNi)_85_Mo_6_Ti_9_, (CoFeNi)_80_Mo_8_Ti_12_, and (CoFeNi)_70_Mo_12_Ti_18_ demonstrated high efficiencies of 97.6, 98.6 and 98.7%, respectively, already in the first minute of reaction.

## Data Availability

The data are available upon request from the corresponding author.
